# Acknowledgment to Reviewers of *Toxins* in 2020

**DOI:** 10.3390/toxins13020088

**Published:** 2021-01-25

**Authors:** Toxins Editorial Office

**Affiliations:** MDPI AG, St. Alban-Anlage 66, 4052 Basel, Switzerland; toxins@mdpi.com

Peer review is the driving force of journal development, and reviewers are gatekeepers who ensure that *Toxins* maintains its standards for the high quality of its published papers. Thanks to the cooperation of our reviewers, in 2020, the median time to first decision was 15.8 days and the median time to publication was 2.8 days. Among the reviewers who reviewed for *Toxins* in 2020, the following two have been selected to receive the “*Toxins* 2020 Outstanding Reviewer Awards” for the timeliness and quality of their review reports in 2020:

Antonets, Kirill S. from All-Russia Research Institute for Agricultural Microbiology (ARRIAM) and St. Petersburg State University, Russia

Rico-Yuste, Alberto from Universidad Complutense de Madriddisabled, Spain

The editors also express their sincere gratitude to the following reviewers for their precious time and dedication, regardless of whether the papers were finally published:
Abad Diaz De Cerio, AnaLi, JingAbd El-Aziz, Tarek MohamedLiaimer, AntonAbdallah, Mohamed F.Liberton, MichelleAboal, MarinaLightfoot, Jorge D.Abou-Mansour, ElianeLimay-Rios, VictorAbraham, AnnLimón, M. CarmenAbraham, Wolf-RainerLin, Cheng-WenAbrahão Nencioni, Ana LeonorLin, GuitingAbrunhosa, LuísLin, Hsin-TangAccoroni, StefanoLin, Shih-HuaÁdám, TölgyesiLindahl, LasseAdamo, ShelleyLingwood, Clifford A.Adams, SolomonLins, LaurenceAdams, VickiLitaker, WayneAdamski, ZbigniewLiu, Biing HuiAdányi, NóraLiu, ChangqiAdewole, DeborahLiu, QianqianAggarwal, MeghaLivaniou, EvangeliaAgha, RamsyLlewellyn, Lyndon E.Agriopoulou, SofiaLluna, Amparo GameroAhel, IvanLo, Samuel Chun-lapAichinger, GeorgLobato-Marquez, DamianAjani, PenelopeLobner, Doug C.Ajigboye, Olubukola O.Locatelli, MarcelloAlassane-Kpembi, ImouranaLoi, MartinaAlbrecht, Eric A.Lopes Fuly, AndréAlcaide, Francisco EspejoLopes, AdrianaAleu, JosefinaLopez Navas, Ana I.Alisaac, EliasLópez-Sanmartín, MonserratAllen, Kerry ClintLou, JianlongAlmeida, MarisaLouzao Ojeda, M. CarmenAlmuhtaram, HuseinLubawy, JanAlonso, EvaLucas, RudolfAlshannaq, AhmadLucke, AnnegretAlter, KatharineLuis, JoséAlvarez-Rodriguez, ManuelLukšić, BorisAmaradhi, RadhikaLuo, MengAmes, Ryan M.Lupette, JosselinAmoriello, TizianaLupo, Maria GiovannaAmorós, AsunciónLuster, Douglas G.Ana, Juan-GarcíaLyukmanova, Ekaterina N.Anas, Andrea Roxanne J.Ma, LinlinAnderluh, GregorMaciejczyk, MateuszAndjelkovic, MirjanaMacKenzie, LincolnAndreev, Dmitriy NikolaevichMackie, RoderickAndreev, Yaroslav A.Macrander, JasonANNIBALLI, FabrizioMaddox, CarolAnsari, RaisMaekawa, MasamitsuAntignani, AntonellaMagalhães, Geraldo S.Antipova, VeronicaMagarlamov, Timur Yu.Antonets, KirillMagnani, MauroAntunes, JoanaMahajan, AbhimanyuAppell, MichaelMahmood, AlfazArakawa, OsamuMaitz, ManfredAraujo, RicardoMajewska, AnnaArez, Ana PaulaMakarova, KaterinaArgiles, AngelMakowska, KrystynaAriño, AgustínMaksimov, Igor V.Armalyte, JulijaMalachová, AlexandraArmengol, RamónMalissiova, EleniArmstrong, GlenMalovichko, Yury V.Artini, MarcoMalysheva, Svetlana V.Asada, KenMammadova-Bach, ElminaAsakawa, ManabuManaila, ElenaAssaf, Jean-ClaudeMancheño, JoséAssunção, RicardoManderville, Richard A.Audsley, NeilManes, JordiAugustyniak, DariaManheim, DerekAustin, JohnManjunath, ManuboluAvantaggiato, GiuseppinaMantle, PeterAvato, PinarosaMantzoukas, SpiridonAvdonin, Pavel V.Manyes, LaraAvelino, AntonioMarabotti, AnnaAwad, MilenaMarchiò, SerenaAxiotis, EvangelosMarchwińska, KatarzynaAyuso, MiriamMargulis, SusanAzarnia Tehran, DomenicoMarie, BenjaminBabica, PavelMarín Ramos, Nagore IsabelBacchiocchi, SimoneMarin, Daniela ElizaBae, Woong JinMarin-Sillue, SoniaBailly, Jean-DenisMarkou, GiorgosBakht, MartinMartchenko, MikhailBakker, MatthewMartínez Del Pozo, ÁlvaroBalawejder, MaciejMartins, CarlaBalestrieri, BarbaraMartins, Ida Sigueko SanoBallot, AndreasMartins, Mariana RenovatoBalogh, EnikőMärtlbauer, ErwinBalogh, KrisztiánMascher-Frutschi, FabioBang, Moon SukMasereeuw, RoosBang, Woo YoungMasiello, MarioBaptista, Mafalda S.Mason, AndrewBarák, ImrichMassy, Ziad A.Barba, Francisco J.Mastanjević, KrešimirBarbano, RichardMastanjević, KristinaBarbier, JulienMastroleo, FeliceBarešová, MagdalenaMasuda, HisakoBaricich, AlessioMasuyer, GeoffreyBarinova, SophiaMatak, IvicaBarisione, ChiaraMathé-Hubert, HugoBarka, Essaid AitMátis, GáborBarth, ValdirMatsui, TaeiBartolotti, MassimoMatsuka, YoshizoBaryeh, KwakuMattei, CésarBasak, Ajit K.Matttila, TapaniBattelli, Maria GiuliaMátyás, CserhátiBaumann, UlrichMauri-Aucejo, Adela R.Bäumer, TobiasMavrikou, SofiaBavikatte, GaneshMazor, OhadBaxter, Simon WadeMazur-Marzec, HannaBeach, DanielMazzantini, DilettaBeard, MatthewMcArdle, StephanieBehnke-Borowczyk, JolantaMcBride, Jere W.Behrends, JochenMcCall, Jennifer R.Bekalu, Zelalem EshetuMcClane, Bruce A.Bel, YolandaMcCormick, Susan P.Belanger, Faith C.Mcgee, David J.Bellasi, AntonioMcKillip, John L.Belloso-Uribe, KepaMcLaughlin, JohnBen-Dov, EitanMcLean, GaryBenedik, MichaelMcMurry, JonathanBenesic, AndreasMebs, DietrichBenson, Janet M.Meggs, William J.Benz, RolandMegighian, AramBera, TapanMegna, MarisaBerestetskiy, AlexanderMehbub, Mohammad FerdousBerhow, Mark A.Mehl, Hillary L.Berrada, HoudaMelville, StephenBerthold-Pluta, AnnaMerrill, A. RodBertin, Matthew J.Mesterhazy, AkosBertuzzi, TerenzioMetcalf, JordanBeug, MichaelMetzinger, LaurentBeuhler, Michael C.Meulenberg, ElineBevc, SebastjanMézes, MiklósBever, Candace S.Michail, VikelisBeyer, WolfgangMichálek, OndřejBhatti, DanishMicheli, LauraBibic, LuckaMiglinas, MariusBierne, HeleneMihaila, Silvia M.Bijak, MichałMilani, MarioBijukumar, DivyaMiller, J. DavidBillerbeck, SonjaMinami, MasaakiBilliald, PhilippeMinervini, FiorenzaBiré, RonelMiranda, Jose M.Blackall, PatrickMir-Sanchis, IgnacioBlackwell, BarbaraMishima, EikanBlanchet, GuillaumeMishra, Santosh K.Blanco, JuanMitchell, MichelaBlasi, JuanMittal, Shivam OmBoarescu, Paul-MihaiMittlmeier, Thomas W. F.Bocian, AleksandraMitulovic, GoranBocian, SzymonMiyoshi, Shin-IchiBocianowski, JanMnif, WissemBoda, FranciscMoar, WilliamBogado Pascottini, OsvaldoModahl, CassandraBokori-Brown, MonikaModica, Maria VittoriaBölcskei, KataModrá, HelenaBondoc, IonelMoha Ou Maati, HamidBongiorno, DafneMohammadi, ShabnamBordes, PatriciaMolgo, JordiBortner, Carl D.Momose, AkishiBoruta, TomaszMonaghan, ThomasBoschetti, ElisaMonika, Waksmundzka-HajnosBosilevac, JosephMonné, MagnusBosland, Maarten C.Monsarrat, PaulBossu, Jean LouisMoon, Byeong CheolBotana, Ana MaríaMoon, Ju-YoungBotelho, Maria JoaoMoon, YuseokBotzenhart, UteMoore, Geromy G.Bouaïcha, NoureddineMoran, YehuBourne, Christina R.Moreira, CristianaBovee, Toine F. H.Mori, TakefumiBowyer, Ruth C. E.Morón-López, JesúsBrancaccio, MariaritaMorton, Steve L.Branchini, AlessioMoschovi, MariaBratek, ZoltánMossialos, DimitrisBraun, VolkmarMostrom, Michelle S.Brčić Karačonji, IrenaMottaghipisheh, JavedBreidbach, AndreasMou, XiaozhenBrewer, MichaelMouchlis, Varnavas D.Brisinda, GiuseppeMowe, MaxineBrodal, GuroMukherjea, DebashreeBrouwer, StephanMüller, ChristianBrown, Amy C.Muller, MarcBrown, Ronald B.Muñoz-Castañeda, Juan R.Brusch, George A.Munuera, PedroBrvar, MiranMurakami, MakotoBryła, MarcinMurata, KazuyaBuchrieser, CarmenMurphy, Caroline S.Burdaspal, PedroMurray, SamBurkin, MaximMurray, ShaunaBurkovski, AndreasMuruganandam, GopinathBurtey, StéphaneMüthing, JohannesBussmann, Rainer W.Mutiga, Samuel K.Buszewski, BogusławNagashima, HitoshiButler-Dawson, JaimeNagel, RaimundButtenschoen, KlausNakahama, Ken-ichiBzducha-Wróbel, AnnaNanaware, PadmaCaballero, ArmandoNanninga, Gerrit B.Caballero, PrimitivoNapierala, MartaCabrera-Guzmán, ElisaNapiórkowska-Krzebietke, AgnieszkaCaceres, IsauraNatsume, MasahiroCai, ShuoweiNaumann, MarkusCaiazzo, ElisabettaNawrot, UrszulaCajado-Carvalho, DanielaNazzal, LamaCalabro, KevinNestorovich, Ekaterina M.Calleros, LauraNeumann, JoachimCallicott, Kenneth A.Ng, Tzi BunCalvete, JuanNgo, Anh H.Cambaza, EdgarNIA, YacineCampas, MonicaNicoletti, RosarioCampos, AlexandreNicosia, AldoČandek, KlemenNielsen, Vance G.Cantaluppi, VincenzoNielsen-LeRoux, ChristinaCantamessa, SimoneNiessen, LudwigCapon, RobertNijman, VincentCappariello, AlfredoNjoku, Dolores B.Caprai, ElisabettaNoda, NaohiroCaprio, MichaelNoy-Porat, TalCarmen, MejíaNunes, Francis Morais FrancoCaronni, SarahNy, AnneliiCaroppo, CarmelaObremski, KazimierzCarqueijeiro, InêsObremski, Kazimierz JanCarroll, RonanOda, MasatakaCaruana, AmandineOgawa, MasahiroCarugo, StefanoOgawa, TomohisaCarvalho, FilipeOh, JaehyeonCasewell, NicholasOkamoto, ShigefumiCasino, PatriciaOkorski, AdamCastro, José Javier FernándezOkumura, ShiroCastro, Mariana S.Okunade, Albert ACatania, SantiagoOlejar, Kenneth J.Cens, ThierryOlejnik, MalgorzataCerasino, LeonardoOliveira Souza, Ana CamilaCeremuga, MichalOliveira, RaquelCerini, ClaireOlli, KalleCerino, FedericaOmur-Ozbek, PinarChan, Michael K.Onda, MasanoriChan, WanOnys’ko, PetroChang, Jer-MingOpiela, JolantaChang, Long-SenOppliger, AnneChapara, VenkatOrlova, OlgaCharlton, ClivelOrr, PhilipChaurasia, Akhilesh KumarOsborne, NickChen, ChenOshiro, NaomasaChen, HuiOstolaza, HelenaChen, JianwuOstrý, VladimirChen, QiOtulak-Kozieł, KatarzynaChen, Shih-ChiehOyama, EtsukoChen, TianbaoOzias-Akins, PeggyChen, YihungPadula, AndrewChen, ZhaoPadula, Matthew P.Cheng, Chun-AnPage, Louise R.Cheng, Luisa W.Painschab, MatthewCherniack, Evan PaulPajek, JernejChernoff, NeilPal, DebjaniChernova, EkaterinaPalanisamy, SatheeshkumarChiang, Hsin-IPałasz, ArturChiang-Ni, ChuanPalma, Mario SegioChijiwa, TakahitoPalumbo, Jeffrey D.Chilaka, CynthiaPanaccione, Daniel G.Chillon, Jean-MarcPandit, HarshulChippaux, Jean-PhilippePannek, JürgenChmielewska, DariaPannella, GianfrancoCho, Jang-HeePantazaki, Anastasia A.Cho, YukoPardali, EvangeliaChoi, Jeong JuneParikesit, Arli AdityaChoque, ElodiePark, Hee-SooChowanski, SzymonPark, JongHoChrismas, NathanPârvu, MarcelChristina, Barja-FidalgoPascale, MichelangeloChu, Pei-MingPascual, Samuel IgnacioChudzinski, Ana MarisaPasternak, GerardChulanov, VladimirPatel, AmarChung, GeehoonPatel, NeepaChung, Se-WoongPaterson, Gavin K.Churro, CatarinaPatkowska, ElżbietaChyau, Charng-CherngPatyra, EwelinaCiarcia, RobertoPaul, AlokCiasca, BiancamariaPawar, Shrikant D.Cimmarusti, Maria TeresaPawlaczyk, KrzysztofCipakova, IngridPawlak, DariuszCirés, SamuelPawlik, AnnaCitores, LuciaPearlman, EricCiufolini, Marco A.Pecorelli, IvanClarke, JohnPedro, SóniaClose, Dan M.Peigneur, SteveClyne, MargueritePelin, MarcoCochran, BlakePellett, SabineCodina, GeorgianaPeltomaa, RiikkaCohen, GeraldPennone, VincenzoCohen-Bouhacina, TouriaPepi, MilvaColloc’h, NathaliePereira, CláudiaComte, KatiaPerera, OmaththageCondon, CiaránPeresin, SoledadConte, AntonellaPeretz, AviCooper, RobinPerez De Souza, LeonardoCórdoba Ramos, María De GuíaPérez-Fernández, MaríaCordoba-Diaz, DamianPérez-Parallé Mera, María LuzCosta, João GuilhermePerez-Pascual, DavidCosta, StefaniaPero, Maria ElenaCosta, Vera MarisaPerrone, GiancarloCostamagna, DomizianaPethő, ZoltánCosta-Rama, EstefaníaPetitpas, Christian M.Cote, ChristopherPetrou, PanagiotaCotella, DiegoPezzolesi, LauraCowardin, CarriePhan, Chin-SoonCozar-Castellano, IrenePhilibert, DanielleCristofori-Armstrong, BenPhillips, JoshuaCrossman, LisaPiccolella, SimonaCruz, CeliaPickett, AndyCruz, FranciscoPicking, William D.Csaba, MáthéPiechowicz, LidiaCuccioloni, MassimilianoPieckova, ElenaCucina, MirkoPiemontese, LucaCui, YuhaiPierron, AlixCunningham, Brady R.Pieszka, MagdalenaCvak, LadislavPietri, AmedeoCzarnota, JoannaPihut, MalgorzataD’Agostino, PaulPikuła, DorotaD’Ambra, IsabellaPincus, SethD’Haese, PatrickPinheiro, JorgeDa Silva, AnaPiotrowska, MałgorzataDa̧browski, MichałPirazzini, MarcoDacher, MatthieuPistocchi, RossellaDal Belo, Cháriston AndréPizoń, MagdalenaDall’Acqua, StefanoPlacido, AntonioDall’Asta, ChiaraPlaczek, RichardDaly, NorellePleadin, JelkaDaly, Seth M.Plötz, MadeleineDam, Chinh Thi MyPócsi, IstvánDamiano, SaraPodobnik, MarjetkaDanforth, Teresa L.Podolska, GrazynaDaniela, JakšićPogrmic-Majkic, KristinaDarius, Hélène TaianaPoirier, Guy G.Darnell, RossPoitevin, StéphaneDatta Gupta, AnupamPoli, MarkDavid, Vlad LaurentiuPolišenská, IvanaDavis, David A.Pollastro, StefaniaDavletov, BazbekPoniedzialek, BarbaraDe Azevedo Calderon, LeonardoPoniedziałek, BarbaraDe Buyzere, Marc L.Poole, DanielDe Castro Figueiredo Bordon, KarlaPoole, RebeccaDe Castro Ramalho, TeodoricoPoór, MiklósDe Cerain, Adela LopezPopham, Holly J.De Haro, LucPortaro, FernandaDe Kimpe, NorbertPosthaus, HorstDe Loor, HenriëttePovey, AndrewDe Marcos Ruiz, SusanaPrakash, DivyaDe Mol, MaartenPratt, ScottDe Rijcke, MaartenPravdin, SergeiDe Simone, SalvatorePredel, ReinhardDe Spiegeleer, BartPrencipe, SimonaDe Stefano, VitaPrentis, PeterDe Witte, PeterPrezoto, Benedito C.Defant, AndreaPriel, AviDegen, GiselaPrigigallo, Maria IsabellaDegola, FrancescaPrimack, WilliamDelanghe, Joris R.Prince, AliceDelcourt, NicolasPrince, Alice S.Dellafiora, LucaPrinciotta, Sarah DeVaulDerman, YagmurProctor, RichardDesprés, LaurenceProft, ThomasDevigili, GraziaPrzybylska-Gornowicz, BarbaraDharmaratne, PriyangaPsaroulaki, AnnaDi Cesare Mannelli, LorenzoPszczółkowska, AgnieszkaDi Nardo, FabioPucca, ManuelaDi Piazza, SimonePucca, Manuela BertoDias, Irundika H. K.Puddick, JonathanDiaz, DuartePuillandre, NicolasDietl, Anna-MariaPulvirenti, AndreaDíez-Quijada, LeticiaPunga, Anna RostedtDilger, Ryan N.Purkrtová, SabinaDing, YousongPuscaselu, RoxanaDinos, GeorgiosPusztahelyi, TündeDiochot, SylvieQuan, Guo-XingDivi, Rao L.Quan, Nguyen VanDjerada, ZoubirQueralt, EthelDobrescu, AmadeusQuereda, Juan JoseDobrowolski, PiotrQuiñones, BeatrizDolly, OliverQuinton, LoicDomashevskiy, Artem V.Radcliff, Fiona JaneDomínguez-Pérez, DanyRádis-Baptista, GandhiDon-Doncow, NicholasRaffaniello, Robert D.Doni, Mihaela BadeaRagucci, SaraDora, DavidRahman, Masmudur M.Dorantes-Aranda, Juan JoséRahnama, MostafaDorner, Martin B.Rakonjac, JasnaDos Santos, Lucilene DelazariRalston, EmilyDos Santos, Regiane RodriguesRam, Angia Sriram PradeepDoucette, GregRamani, KomalDreher, TheoRamesh, Tennore M.Drewnowska, JustynaRampersad, SephraDubbini, AlessandraRasetti-Escargueil, ChristineDucancel, FrédéricRattazzi, MarcelloDucatelle, RichardRaymond, BenDucreux, SylvieRaymond, JosetteDuke, Stephen O.Reddy, PriyankaDunbar, JohnReguera, BeatrizDuncan, KatherineReich, JohannesDunisławska, AleksandraReis-Costa, PedroDunlop, RachaelReitzel, Adam M.Dunyach-Remy, CatherineRekand, TiinaDurán-Patrón, RosaRementeria, AitorDurán-Riveroll, LorenaRemigante, AlessiaDuranti, ClaudiaRenaud, JustinDurazzo, AlessandraRepka, SariDurek, ThomasResiere, DaborDutertre, SebastienRestivo, DomenicoDutta, SanjuctaRestivo, Francesco MariaDuval, DavidReuter, TimDziga, DariuszRho, Nark-KyoungEberhardt, OlafRiboldi, Giulietta M.Ebert, Timothy A.Richardson, Michael K.Eble, JohannesRichardson, Simon C. W.Edwards, Adrianne N.Rico-Yuste, AlbertoEfremenko, ElenaRigano, FrancescaEibl, Martha M.Righetti, LauraEkino, KeisukeRiva, FedericaEl Far, OussamaRobertson, AlisonElble, Rodger J.Robinson, Samuel D.Eldeeb, MohamedRocchetti, Maria TeresaEleopra, RobertoRockenfeller, PatrickElls, TimothyRodemann, BerndEl-Mallakh, RifRodríguez, AliciaEl-Seedi, Hesham R.Rodríguez, Armando AlexeiEltzov, EvgeniRodríguez-Estévez, VicenteEndo, HarukaRohrlack, ThomasErnst, MargotRojo, Maria AngelesEspiña, BegoñaRolland, Jean-LucEsteves, TeresaRolston, JohnEtxebeste, OierRos, UrisEvans, Tim J.Roscini, LucaEveraert, KarelRosic, GvozdenEvidente, AntonioRosier, Peter F. W.Faassen, ElisabethRoss, Kenneth G.Fabbri, AlessiaRossetto, OrnellaFabioux, CarolineRossi, FilippoFabiszewska, AgataRossignoli, Araceli EscudeiroFaccioli, Lucia HelenaRostoker, GuyFagerquist, Clifton K.Rothkoetter, Hermann-josefFalconer, Ian R.Roué, MélanieFalquet, LaurentRouge, PierreFan, YongfengRoutledge, MichaelFanizzi, Francesco PaoloRoutledge, Michael N.Faquim-Mauro, Eliana L.Rowe, Matthew P.Faraone Mennella, Maria RosariaRoyal, Joshua M.Farka, ZdeněkRoy-Lachapelle, AudreyFasullo, MichaelRubio, SoledadFaustini, MassimoRubiolo, JuanFébel, HedvigRueter, JohnFeig, AndrewRuiu, LucaFelicori, LizaRummel, AndreasFeodorova, YanaRusak, TomaszFernandes, Ana S.Russo, GiulioFernandes, Luís Gustavo RomaniRuvindy, RendyFerran, AudeRydberg, SaraFerré, JuanRyu, Ji-wonFerrer, EmiliaRzymski, PiotrFerreras, José M.Sachkova, MariaFerreras, José MiguelSadhasivam, SudharsanFerret-Bernard, StéphanieSadok, IlonaFilep, János G.Saitoh, KenjiFilipeanu, CatalinSalazar, EmelynFine, DanielSaleh, MazenFinkel, Alan G.Sales, EsterFinlayson, HeatherSallée, MarionFinol-Urdaneta, Rocio K.Salvatore, Maria MichelaFišer, ŽigaSamdal, IngunnFitzgerald, DavidSamson, Rob A.Flannery, BrennaSanchez, AnaFleites, Laura A.Sánchez-del-Campo, LuisFlorens, NansSandomenico, AnnamariaFlores, CintiaSanny, CharlesFlores-Guerrero, Jose L.Sant’anna, MorenaFlórez, Laura V.Santini, AntonelloFogle, JonathanSantos-Filho, NorivalFolli, ClaudiaSantovito, ElisaFontaine, FlorenceSanz, AnaForstova, JitkaŠarkan, BojanForte, ElviraŠarkanj, BojanFozo, ElizabethSarkes, Deborah A.Fraguas-Sánchez, Ana IsabelSarzani, RiccardoFranciosa, GiovannaSatake, MasayukiFrank, DaraSato, EiichiFreitas, MarisaSato, EmikoFrevert, JuergenSatta, Cecilia T.Frey, JoachimSauer, John-DemianFridolin, IvoSaurav, KumarFrisan, TeresaSavadova, KsenijaFrosini, SianSavoie, Jean-MichelFujinaga, YukakoSawbridge, Timothy I.Fujita, MasakiSayyari, AminFukuyama, TomokiSchaarschmidt, SaraFuly, AndréSchäfer, KatrinFurukawa, TomohiroSchardl, Christopher L.Gagat, MaciejSchininà, Maria EugeniaGagiu, ValeriaSchmidt, GudulaGago-Martínez, AnaSchmidt, HerbertGajęcki, MaciejSchmidt, JustinGalan, JacobSchmidt, MartinGalani, Joseph Hubert YamdeuSchmidt, ThomasGalić, VlatkoSchmitt-Keichinger, CorinneGall, BrianSchroeder, ChristinaGallardo, EugéniaSchröper, FlorianGallo, AntoniaSchrum, AdamGamil, AmrSchurgers, Leon J.Gandolfi, MarialuisaSchwartz-Zimmermann, Heidi ElisabethGarcía, GabrielaSchweiger, WolfgangGarcía, TaniaSciani, JulianaGarcía-García, AinaScrano, LauraGarcía-Linares, SaraScudiero, OlgaGarcía-Ortega, LucíaSedova, IrinaGardner, Dale R.Seger, AndreasGarganese, FrancescaŠegvić Klarić, MajaGastaldello, StefanoSelby, KatjaGauer, StefanSelva, LauraGauthier, ThierrySelvaraj, ArokiyarajGawel, KingaSeo, Jung-KilGe, ZhongmingSerafín, VerónicaGeisler, ChristophSethi, GautamGembillo, GuidoSeubert, Erica L.Gerard, NormaShahi, Shailesh K.Gerardino, AnnamariaShen, JianqiangGergova, RainaShenkarev, Zakhar O.Gerhard, RalfShibata, HirokiGerin, DonatoShimamura, YukoGhirga, FrancescaShirato, KenGhosh, SumitShishido, Tânia K.Giannantoni, AntonellaShnyder, SteveGiannuzzi, LedaShoguchi, EiichiGiansanti, FrancescoShrestha, NishaGibble, CorinneShridhar, SurekhaGibbs, H. LisleSidiropoulos, ChristosGientka, IwonaSieroslawska, AnnaGil Serna, JéssicaSierra, Josep M.Gill, ChandlerSilaghi, Ciprian N.Gilles, NicolasSilva, AnjanaGillet, DanielSilva, Christopher J.Gilliam, Lyndi L.Simm, RogerGiorni, PaolaSimões, João Carlos CaetanoGirard, BrigitteSimon, StephanieGirbes, TomasSims, GeraldGirolami, FlaviaŠindlerová, LenkaGirolamo, Annalisa DeSingh, BalGladan, Živana NinčevićSingh, PummiGlibowski, PawełSingharoy, AbhishekGoldman, EllenSinha Ray, ShresthaGoldstein, JosephineSintsova, OksanaGomes, Luciana CalheirosSionov, EdwardGomez, AntonioSivakumar, SushamaGómez-Baena, GuadalupeSix, IsabelleGomis-Cebolla, JoaquinSizova, OlgaGoncalves, CarlosSkladanka, JiriGonçalves, Luis Roberto De CamargoSlama, PetrGonkowski, SlawomirŚledziński, TomaszGonzález-Peñas, ElenaŚliwińska-Wilczewska, SylwiaGorson, JulietteSliwinski, TomaszGosetti, FabioŚliżewska, KatarzynaGraham, KerrSmania, NicolaGrajewski, JanSmeu, IrinaGramignoli, RobertoSmith, JocelynGrass, GregorSmith, Zacharias J.Grebinyk, AnnaSnauwaert, EvelienGreco, GraziaSnelling, WilliamGreen, A. ChristopherSnow, Nicholas H.Green, BenSoares, Antonio GarciaGreen, MichaelSoares, Celia MariaGrela, PrzemysławSogawa, NorioGrenda, TomaszSolcan, CarmenGrieco, FrancescoSolis, Itzue CaviedesGrimaldi, GiovannaSolovchenko, AlexeiGromadzka, KarolinaSomma, StefaniaGrossi Bovi Karatay, GrazieleSong, JiajiaGroth, Christopher L.Soulage, Christophe O.Gu, ZhenSousa, SandraGuerre, PhilippeSovadinova, IvaGuimarães, Jorge A.Španič, ValentinaGulic, TamaraSpassov, SashkoGulsevin, AlicanSpeeckaert, MarijnGundert-Remy, UrsulaSpizzirri, Umile GianfrancoGuo, JingshuSprawka, IwonaGuthridge, Kathryn M.Stadio, Arianna DiGutierrez, ClaudeStanciu, OanaGutierrez, SantiagoStanek-Tarkowska, JadwigaGutmann, MarcusStasiewicz, Matthew J.Guzik, MaciejStatsyuk, Natalia V.Habibu, MugerwaStegelmeier, BryanHachani, AbdouStegmayr, BerndHaesaert, GeertStenmark, PalHalassy, BeataŠtěpánová, HanaHall, Pamela R.Stępień, ŁukaszHallegraeff, Gustaaf M.Stern, DanielHallen-Adams, Heather E.Stessl, BeatrixHaller, Steven T.Stewart, George C.Hallsworth, J. E.Stewart, Linda D.Hamamichi, ShuseiStines-Chaumeil, ClaireHamano, HirofumiStixova, LenkaHammes, Mary S.Stommel, ElijahHan, Jong WonStowell, Michael H. B.Hanna, RamyStoyneva-Gärtner, MayaHanna, Ramy M.Strekalova, TatyanaHans, GuyStrickland, JasonHarada, Ken-ichiStrobel, BjarneHarijan, Rajesh K.Stromberg, ZacharyHarke, MatthewStrzalkowski, NickHarm, StephanStuper-Szablewska, KingaHarris, LindaSuarez, DimasHarris, Ted D.Subbaraman, Meenakshi S.Harris, Thomas M.Suess, BeatrixHasegawa, MakotoSugita-Konishi, YoshikoHassan, ErrolSułek, AlicjaHassan, YasserSulewska, HannaHawlitschka, AlexanderSumarah, Mark WHayashi, Mirian Akemi FuruieSummers, MatthewHe, QinshuiSun, HongminHealy, Helen G.Sun, XingminHegaret, HeleneSun, YvonneHeir, Gary M.Sundheim, LeifHellinger, RolandSung, Wang ChouHellman, AmySuntravat, MontamasHellmann, NadjaSuo, ReiHénaut, LucieŠuput, DušanHenderson, BrandonSurmann, KristinHeneberg, PetrSurup, FrankHernández-León, AlejandroSusca, AntoniaHerrera, MartaSuyama, KeitaroHerrin, DavidSuzuki, ShigekatsuHesselson, DanielSuzuki, TakehiroHeuck, AlejandroSuzuki, ToshiyukiHibbs, RyanSwamy, PrashantHietaniemi, VeliSwan, Sarah C.Hlavová, KarolinaSycha, ThomasHo, MengfeiSzabó, AndrásHofer, Matthias D.Szajwaj, MonikaHolmar, JanaSzczerba-Turek, AnnaHolzmann, CarstenSzulc, JustynaHoogenboom, RonTabler, TomHort, VincentTadahiro, SuzukiHossler, Eric W.Taguchi, KenseiHoward, Ileana M.Tai, Chin-HsienHruska, ZuzanaTakata, TomoakiHsu, Bang-geeTakumi, ShotaHsu, Jih-TayTalà, AdelfiaHu, ChenlinTan, YanglanHu, Kyung-SeokTang, Ho ManHuang, I-HsiuTangni, Emmanuel K.Huang, San-YuanTaraseviciene, ZivileHuang, Tur-FuTarng, Der-CherngHughes, ScottTartaglione, LucianaHuguet, AntoineTashima, ToshihikoHumpage, AndrewTasoulis, TheoHungerford, NatashaTatsuno, RyoheiHuntley, Jason F.Tatulian, Suren A.Hutorowicz, AndrzejTaub, MaryHuzzatul Mursalin, MdTavcar Kalcher, GabrijelaIammarino, MarcoTaylor, MatthewIanieri, GiancarloTchórzewski, MarekIatsenko, IgorTerzi, ValeriaIekhsan, OthmanTeschke, RolfIglesias, Maria Del RosarioTessaro, FrancescaIijima, NoriakiTeter, KenIkeda, YasumasaTetreau, GuillaumeIkegaya, NaokiThanki, Anisha MahendraIkonomopoulou, MariaTharmalingam, NagendranIkushiro, Shin-ichiTheodorakis, PanagiotisIlia, GheorgheTheuerkauf, JörnImai, YasuyukiThiel, TeresaInagaki, HidetoshiThomas, María del MarInoue, SeijiThomas, SethIoannis, HatzianestisTian, FurongIorga, BogdanTichaczek-Goska, DorotaIrigoyen, AngelTietze, DanielIshihara, JunkoTikhonova, IrinaIsola, GaetanoTiminszky, GyulaItoi, ShiroTinker, JulietteIzumiya, HidemasaTocmo, RestitutoJablonska, EwaToedebusch, ChristineJackson Woo, Chit ShingToiu, AncaJacobs-Cachá, ConxitaTolosa, JosefaJaime, RamonToma-Fukai, SachikoJakubczyk, DorotaTomaru, YujiJames, MargaretTomassini, LambertoJamet, AnneTomczyk, ŁukaszJaneczko, MonikaTonello, FiorellaJanšta, PetrTorović, LjiljaJaradat, NidalTorres, JaumeJedrejek, DariuszTorres-Russotto, DiegoJedziniak, PiotrTrainer, VeraJedziniak, Piotr JanTrammell, SamuelJeßberger, NadjaTreilhou, MichelJeyanathan, JeyamalarTrif, MonicaJia, YingTrim, Steven A.Jiménez-Tenorio, ManuelTrumble, John T.Jin, ZhaoTsai, Inn-HoJobling, PhilTsai, Jaw-ShiunJog, MandarTsai, Yien CheJohn, RohanTsakiris, Ioannis N.Johnson, EricTsuboi, NaotakeJohnson, Shannon L.Tsumuraya, TakeshiJohnson, Stephen B.Tsuneda, SatoshiJoniak, TomaszTsunoda, MakotoJos, ÁngelesTufarelli, VincenzoJosić, ĐuroTufféry, PierreJost, Wolfgang H.Tuffs, Stephen W.Joung, Hyou-ArmTurnbull, BruceJovaišienė, JurgitaTurner, ClaireJuan García, CristinaTwarużek, MagdalenaJuhna, TalisTwiss, Michael R.Jung Park, HueTzeng, Shiang-JongJung, Tae SungTzika, EleniJurenas, DukasUde, ChristianJurkiewicz, PiotrUebbing, SeverinJuś, KrzysztofUegaki, RyuichiKaas, Quentin K.Ulrich, SebastianKadowaki, DaisukeUpdegrove, Taylor B.Kahl, Barbara C.Urquhart, Bradley L.Kahlert, StefanUtkin, YuriKajjumba, George WilliamUwai, KojiKakabakos, SotiriosVale, CarmenKalb, Suzanne R.Vale, PauloKalhotka, LiborValenzuela, StellaKalogianni, DespinaValério, ElisabeteKaloudis, TriantafyllosVamanu, EmanuelKameneva, PolinaVan Ballegooijen, HanneKaminski, ArielVan Den Broeck, WimKaminski, Tomasz W.Van Den Heever, Johan P.Kamm, ChristophVan Den Heuvel, LambertusKamruzzaman, MuhammadVan Der Schans, MarcelKamzolova, SvetlanaVan Ooij, ChristiaanKananavičiūtė, RūtaVan Selms, Maurits K. A.Kanerva, MerviVannucchi, Maria-GiulianaKang, Jeong-HyunVanzant, Eric S.Kang, TaejoonVardaka, ElisabethKant, ShivaVasconcelos, Teresa Maria Pinto Coelho AmadoKapolos, JohnVašková, JankaKaraüzüm, HaticeVassiliadis, SimoneKarlson, BengtVattepu, RaviKarp, Barbara IllowskyVaughan, Martha M.Karpiński, MirosławVavitsas, KonstantinosKarpinski, Tomasz M.Vázquez-Ibar, José LuisKarus, AvoVeiga, SilvioKataoka, HiroshiVeleeparambil, ManojKatikou, PanagiotaVelickovic, DusanKayser, Reilly R.Velíšek, JosefKazandjian, TalineVelotta, RaffaeleKędzierska, BarbaraVenancio, ArmandoKehl, AlexanderVerbeke, FrancisKeller, UllrichVerdes, AidaKerns, DavidVerma, ArjunKeyler, Dan E.Versino, ElisabettaKikuta, ShingoVidal, ArnauKim, Baik-HoVieira, Gustavo Henrique CalazansKim, Byoung SikVietinghoff, Sibylle VonKim, Hee-JinViggiano, DavideKim, HoonVilariño, NataliaKim, Jae-SeokViñas, MiguelKim, Kyu-BongVismarra, AliceKim, Seong TaekVogel, HeikoKim, Seong-GonVuko, ElmaKim, WoojinVyviurska, OlgaKimura, TadashiWagg, AdrianKirienko, NatashaWagner, Nicole D.Kiseleva, MariyaWaliullah, SumyyaKiss, DavidWalker, AndrewKlähn, StephanWalker, MichaelKlich, DanielWalkowiak, SeanKlompen, Anna M. L.Walkowiak-Nowicka, KarolinaKlotz, James L.Wallace, Kenneth N.Kluess, JeannetteWalter, UweKnaga, SebastianWang, Chung-ChengKnobloch, Thomas J.Wang, HaiKobayashi, NobumichiWang, JeffreyKoch, MatthiasWang, Shu-HueiKodali, MaheedharWarzecha, TomaszKoelman, JohannesWaskiewicz, AgnieszkaKoksharova, Olga A.Watanabe, HiroshiKolackova, IvanaWatanabe, RyuichiKolawole, OluwatobiWawrzyniak, JolantaKoliesnik, IevgenWeaver, AlexandraKollewe, KatjaWeaver, KeithKoppe, LaetitiaWeber, BarbaraKorch, Shaleen B.Weber, David S.Kordowitzki, PawełWee, JosephineKorhonen, Hannu J. T.Wegrzyn, AlicjaKorponai, JanosWegrzyn, GrzegorzKosicki, RobertWeise, David R.Kosnik, MitjaWeiser, Douglas C.Kostić, Aleksandar Ž.Wejnerowski, ŁukaszKőszegi, TamásWelch, KevinKoudelka, Gerald B.Weller, Michael G.Kovac, JasnaWells, TracyKovač, TihomirWenda-Piesik, AnnaKovbasnjuk, OlgaWeng, AlexanderKowalska, KarolinaWest, FrederickKoyack, MarcWesthoff, Mike-AndrewKozachok, SolomiiaWestphal, AndreasKozak, AnnaWharton, Rebekah E.Kozlov, SergeyWhite, BlanaidKranzler, MarkusWhitlock, Brian K.Krauss, JochenWhittington, CarlKretschmer, DorotheeWichelen, Jeroen VanKroc, MagdalenaWidemann, EmilieKrock, BerndWiegand, ClaudiaKrol, EwelinaWiesner, JacquelineKrstanović, VinkoWiewióra, BarbaraKrug, Hollis E.Wilcox, ChristieKrzebietke, Sławomir JózefWilk-Wozniak, ElzbietaKsieniewicz-Woźniak, EdytaWilliams, E. MarkKubala, LukášWillis, AnusuyaKubatzky, KatharinaWilson, HeatherKubo, TaiWilson, ThomasKudryashov, Dmitri S.Winkel, KenKuhlmann, F. MatthewWinkler, Duane D.Kuhnert, EricWissel, JörgKuhn-Nentwig, LuciaWolfe, DanielKulma, MagdalenaWong, ChristianKumamoto, EiichiWoodhouse, JasonKumar, AkhileshWorobo, Randy W.Kumar, GauravWöstemeyer, JohannesKumar, PavanWoźny, MaciejKumar, RajWree, AndreasKumar, SujeetWright, Denis J.Kunova, AndreaWrzosek, MałgorzataKunsági-Máté, SándorWu, Ming-JiuanKuo, Mei-ChuanWu, XiaosaKuo, Tsung-RongWu, YunkeKuramitsu, KazumuWürzner, ReinhardKurka, OndrejXing, MinliKurtović, TihanaXiong, YanqiongKuzmin, AntonXu, JinKuźniar, AgnieszkaYagi, NaotoKwiatkowski, PawelYamaguchi, YoshihiroLaboha, PetraYamakawa, HiroyukiLacey, JakeYamamoto, AkihikoLackner, ReinhardYamamoto, SuguruŁaczmański, ŁukaszYan, MengLaGrow, Austin L.Yanda, MuraliLahondere, ChloeYang, BoLai, SilviaYang, Chung ShuLalak-Kańczugowska, JustynaYang, JunhuaLam, Kwok HoYang, Yi-YuanLami, AndreaYanshole, Lyudmila V.Lamothe, ShawnYasin, SohailLancaster, PhillipYasuda, ShinLandschoot, SofieYasuhiko, KogaLangenberg, JanYelko, Rodríguez-CarrascoLanot, AntoineYemmireddy, Veerachandra K.Lanzanova, ChiaraYin, KathleenLassudrie, MalwennYoon, Kyungjae-andrewLatek, DorotaYoshihisa, TomiokaLattanzio, Veronica Maria TeresaYoshinari, TomoyaLavrenčič, AndrejYotsu-Yamashita, MariLax, AlistairYu, JianmeiLazarovici, PhilipYu, Yu-HsiangLazzarini, EdoardoYudushkin, IvanLeal, FernandoZacchia, MiriamLeanza, GiampieroZádor, ErnőLeao, JoséZambelli, VanessaLeão, Pedro N.Zancolli, GiuliaLebogang, LesediZaragoza, WilliamLeclère, LucasZarfel, Gernot E.Lee, ChanZargar, AminLee, Christine H. C.Zatsepin, TimofeiLee, Hsin-ChenZeigler, DanielLee, Min-HoZeleny, ReinhardLee, Moo-SeungZervou, Sevasti-KiriakiLee, SangheeZewdie, Ephrem TakeleLee, Tse-MinZhang, KaiLeggieri, Marco CamardoZheng, Cai-MeiLelario, FilomenaZheng, JieLemichez, EmanuelZherdev, Anatoly V.Leo, Jack ChristopherZhou, Carol L. EcaleLeonardi, AdrijanaZielonka, ŁukaszLeonelli, FrancescaZieniuk, BartłomiejLepiarczyk, EwaZimba, PaulLepoutre, AlexandraZingali, Russolina BenedetaLetek, MichalZingli, RussolinaLevesque, AmélieZsarnovszky, AttilaLewczuk, BogdanŻuk, MagdalenaLewin, MattZuliani, Juliana PavanLewis, RichardZupko, IstvanLi, DeyaoZupo, ValerioLi, Dongying


